# Follow-Up Timing After Discharge and Suicide Risk Among Patients Hospitalized With Psychiatric Illness

**DOI:** 10.1001/jamanetworkopen.2023.36767

**Published:** 2023-10-09

**Authors:** Song Ee Che, Yeong Geun Gwon, Kyoung-Hoon Kim

**Affiliations:** 1 Review and Assessment Research Institute, Health Insurance Review and Assessment Service, Wonju, South Korea; 2 Department of Health Administration, Kongju National University, Gongju, South Korea

## Abstract

**Importance:**

Although early outpatient follow-up after psychiatric inpatient discharge may prevent suicide, the association between early follow-up care and a reduced risk of suicide after discharge has not been fully explored.

**Objectives:**

To investigate outpatient follow-up care after psychiatric inpatient discharge and determine whether the timing of the first outpatient follow-up is associated with a reduced risk of suicide.

**Design, Setting, and Participants:**

This population-based, retrospective cohort study used the National Health Claim Database from 2017 to 2018 in Korea. Patients were observed until December 31, 2021, to confirm the occurrence of suicide. The study population included all patients aged 18 years or older who were newly admitted to hospitals with psychiatric illness except for dementia from January 1, 2017, to December 31, 2018. Statistical analysis was performed from January to May 2023.

**Exposure:**

The timing of the first mental health outpatient care follow-up within 30 days after discharge.

**Main Outcomes and Measures:**

The outcome of interest was suicide after hospital discharge. The timing of the first follow-up visit was observed within 30 days after discharge. The Cox proportional hazard model was used to explore the association between the risk of suicide and the timing of outpatient follow-up.

**Results:**

Of the 76 462 patients admitted to hospitals from 2017 to 2018, 225 (52.6%) were male; 21 313 (27.9%) had a primary diagnosis of substance use disorder, 17 608 (23.0%) had schizophrenia, and 15 018 (19.6) had depression; mean (SD) age was 46.4 (16.3) years. A total of 49 319 patients (64.5%) received follow-up outpatient care within 30 days of discharge. The mean (SD) follow-up period was 30.8 (20.2) months, and 1536 patients died of suicide during the study period. The hazard ratio for suicide risk in patients who received outpatient care within 7 days compared with those who did not receive any care within 30 days after discharge was 0.82 (95% CI, 0.80–0.83). The earlier the time of outpatient follow-up care, the lower the risk of suicide for those with substance use disorder, schizophrenia, bipolar disorder, and depression.

**Conclusions and Relevance:**

In this cohort study of patients with psychiatric illness, early follow-up outpatient care after discharge was associated with a lower risk of suicide. These results suggest that those at high risk of suicide during hospitalization need intensive follow-up immediately after discharge.

## Key Points

**Question:**

Is the timing of the first outpatient follow-up after psychiatric inpatient discharge associated with a lower risk of suicide?

**Findings:**

In this cohort study using a nationwide population-based database of 76 462 patients, earlier outpatient care was significantly associated with lower risk of suicide, particularly for patients with substance use disorder, schizophrenia, bipolar disorder, and depression.

**Meaning:**

These findings suggest that more intensive follow-up during the period immediately after discharge is needed to prevent suicide among patients with psychiatric illness.

## Teaser

This cohort study examines whether the timing of the first outpatient follow-up after psychiatric inpatient discharge is associated with a reduced risk of suicide.

## Introduction

The risk of suicide immediately after discharge from a psychiatric hospital is considerably high.^1^ According to a meta-analysis, the pooled estimates of the suicide rate in the first week and month of discharge were 2950 and 2060 suicides per 100 000 person-years, respectively.^2^ A recent Korean study reported that the standardized mortality ratio for suicide within 30 days after psychiatric inpatient discharge was 66.8 compared with the general population between 2016 and 2018.^3^ In order to prevent suicide after discharge from a psychiatric hospital, early follow-up care after discharge is strongly recommended, as it provides an opportunity for intervention or management and to reevaluate whether patients may be at risk for suicide.^4,5,6,7^

Several studies have reported an association between outpatient follow-up after psychiatric inpatient discharge and health outcomes. Among adults enrolled in North Carolina Medicaid who were discharged with depression and schizophrenia, follow-up within 7 days of discharge was associated with greater medication adherence compared with no follow-up within 30 days.^8^ A retrospective cohort study of schizophrenia and bipolar disorder found that outpatient follow-ups within 30 days after discharge were associated with slightly lower readmission risk.^9^ Another study revealed that patients who received timely follow-up visits had lower readmission rates in Japan^10^ and the US.^11^ Some studies examining the association between early outpatient follow-up after discharge and suicide risk have been published. A recent population-based study reported that the risk for suicide after psychiatric hospitalization was low among youths aged 10 to 18 years who had an outpatient visit within 7 days of discharge.^12^ Similarly, a cohort study using the Veterans Affairs administrative database found that patients who received at least 2 outpatient care visits in only 1 of 3 two-month periods within 6 months after discharge from a psychiatric hospital were at increased risk for suicide.^13^

Outpatient follow-up visit after psychiatric inpatient discharge has been used as a quality indicator to assess the performance of the mental health system,^14^ as it is considered important to the reduction of the risk of suicide. The National Committee for Quality Assurance in the US regularly reports the rate of follow-up within 7 or 30 days after discharge from a psychiatric hospital^15^ to provide the basis for establishing accountability of high-quality care.^16^ Moreover, the National Confidential Inquiry into Suicide and Safety in Mental Health and National Institute for Health and Care Excellence (NICE) recommends a follow-up period of 2 to 3 days and 7 days, respectively, for patients discharged from the hospital with psychiatric illness.^17,18^ However, the association between early follow-up care and a reduced risk of suicide after discharge has not been fully explored. This study aims to investigate outpatient follow-up care after psychiatric inpatient discharge and examine whether the timing of the first outpatient follow-up within 30 days after discharge was associated with a lower risk of suicide using a population-based database.

## Methods

### Ethical Considerations

This cohort study was approved by the institutional review board of the Health Insurance Review and Assessment Service with a waiver of informed consent because deidentified data were used. This study followed the Strengthening the Reporting of Observational Studies in Epidemiology (STROBE) reporting guideline.^21^

### Data Source

This population-based, retrospective cohort study used the National Health Claim Database (NHICD) from the Health Insurance Review and Assessment Service. The NHICD included demographic characteristics and health care services, such as prescription and examination, for all patients.^19^ The NHICD was linked with the National Death and Cause Database (NDCD) from the Statistics Office in Korea using unique patient identifiers to identify information on the cause of death.

### Study Population

This study included all patients aged 18 years or older who were newly admitted to hospitals with psychiatric illness, except for dementia, from January 1, 2017, to December 31, 2018. In order to include a homogeneous population, those who had been admitted within the previous 3 years based on index admission were excluded. Patients were also excluded from the study population if they died by suicide or other causes during the hospital stay or on the day of discharge (Figure).

**Figure.**
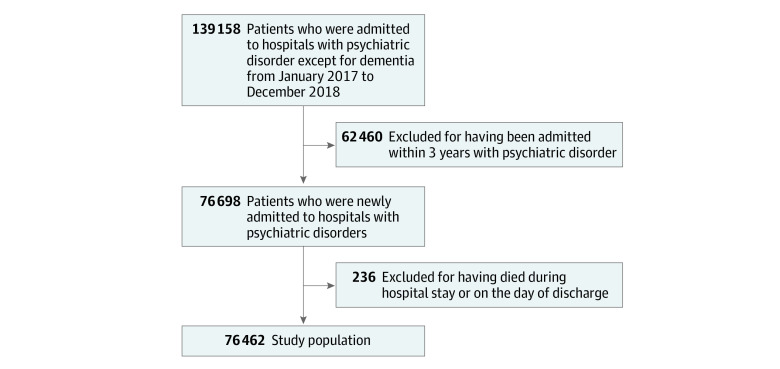
Flowchart of Patients Included

### Outcome Measures and Variables

The outcome of interest was suicide after psychiatric inpatient discharge. Suicide was identified by *International Statistical Classification of Diseases and Related Health Problems, Tenth Revision (ICD-10)* codes X60 to X84 as the primary cause of death by the NDCD. To separate exposure and follow-up periods, patients were observed from 30 days of discharge until December 31, 2021, to confirm the occurrence of suicide.

The exposure of interest was the timing of the first outpatient follow-up of patients with psychiatric illness within 30 days after psychiatric discharge from hospitals. It was categorized as occurring within 7 days, 8 to 14 days, 15 to 30 days, or none in 30 days after discharge. As potential covariates, this study included age at hospital discharge (categorized as 18-29, 30-39, 40-49, 50-59, 60-69, and ≥70 years), sex, type of health insurance (health insurance and Medicaid), diagnostic group, history of outpatient care for psychiatric illness, and Charlson comorbidity index (CCI). The diagnostic groups were classified based on the prevalence of primary diagnosis: substance use disorders (*ICD-10*: F1x.x), schizophrenia (*ICD-10*: F2x.x), depression and other mood disorders (*ICD-10*: F32.x, F33.x, F34.1), anxiety and stress disorders (*ICD-10*: F4x.x), and other mental disorders (*ICD-10*: remainder of F10.x to F99.x). The history of outpatient care and CCI was measured by screening health service utilization for the previous 3 years based on index admission. Comorbidity assessment is essential in accounting for differences in a patient’s health status.^20^ The CCI is a widely used comorbidity measurement in health research using claims data.

### Statistical Analysis

Patients’ demographic characteristics were presented as descriptive statistics, which included frequency, percentage, mean, and SD. The comparisons between the demographic characteristics and the timing of outpatient follow-up within 30 days after discharge were tested using analysis of variance for continuous variables and χ^2^ tests for categorical variables. Suicide rates per 100 000 person-years were defined as the number of suicides divided by the amount of person-years, multiplied by 100 000, and the corresponding 95% CIs were reported. The Cox proportional hazard model was used to explore the association between the risk of suicide and the timing of outpatient follow-up after adjusting for age, sex, CCI, type of health insurance, and history of outpatient care for psychiatric illness, estimated adjusted hazard ratio (HR), and 95% CI. To separate exposure and follow-up periods, patients were observed from 30 days of discharge until December 31, 2021, and those who were either readmitted or died from other cause of death were considered to be censored. All statistical analyses were performed from January to May 2023 with SAS Enterprise Guide 7.1 (SAS Institute), and 2-sided *P* < .05 was considered statistically significant.

## Results

### Demographic Characteristics of the Study Population

Among the 76 462 patients admitted to hospitals from 2017 to 2018, 40 225 (52.6%) were male; 21 313 (27.9%) had a primary diagnosis of substance use disorder, 17 608 (23.0%) had schizophrenia, and 15 018 (19.6) had depression; mean (SD) age was 46.4 (16.3) years. Of 76 462 patients with no previous psychiatric hospitalization, 48 218 (63.1%) had a CCI of 1 or higher, and 55 989 (73.2%) had previous experience in outpatient care for psychiatric illness.

There were 49 319 patients (64.5%) who received an outpatient follow-up within 30 days of discharge; 26 489 (34.6%) received an outpatient follow-up within 7 days of discharge, 15 264 (20.0%) within 8 to 14 days, and 7566 (9.9%) within 15 to 30 days. Of all the age groups, the rate of receiving a follow-up visit within 30 days of discharge was the highest in the age group of 18 to 29 years (77.9% [11 618 of 14 917]), and it was lowest among patients aged 70 years or older (54.8% [3292 of 6011]). The follow-up visit rate for men within 30 days of discharge was 57.1% (22 970 of 40 225), lower than that for women (72.7% [26 349 of 36 237]). The rate of receiving follow-up visits for those with health insurance was 65.8% (41 730 of 63 437), higher than for those with Medicaid (58.3% [7589 of 13 025]). The lower the CCI, the lower the rate of follow-up visits within 30 days. The follow-up visit rate for patients with bipolar disorder was 84.7% (5818 of 6871), which was the highest among psychiatric illness, followed by follow-up visits for those with depression (77.9% [11 695 of 15 018]), schizophrenia (75.4% [13 278 of 17 608]), anxiety disorder (63.6% [6977 of 10 964]), and substance use disorder (41.4% [8819 of 21 313]). The rates of receiving a follow-up visit within 7 days after discharge were higher than other timings of visiting the outpatients across all psychiatric illness, followed by receiving a follow-up visit within 8 to 14 days. Table 1 depicts the patients’ characteristics.

**Table 1.** Demographic Characteristics of the Study Population

Variables	Patients, No. (%)						*P *value
	Total	No follow-up visit	Outpatient care visit after discharge				
			Subtotal	≤7 d	8-14 d	15-30 d	
Total	76 462 (100.0)	27 143 (35.5)	49 319 (64.5)	26 489 (34.6)	15 264 (20.0)	7566 (9.9)	NA
Age, mean (SD), y	46.4 (16.3)	50.1(15.5)	44.4 (16.3)	43.2 (16.4)	45.0 (16.3)	47.3(15.6)	<.001
18-29	14 917 (19.5)	3299 (22.1)	11 618 (77.9)	7002 (46.9)	3391 (22.7)	1225 (8.2)	<.001
30-39	11 118 (14.5)	3175 (28.6)	7943 (71.4)	4509 (40.6)	2370 (21.3)	1064 (9.6)	
40-49	16 091 (21.0)	5847 (36.3)	10 244 (63.7)	5260 (32.7)	3207 (19.9)	1777 (11.0)	
50-59	18 029 (23.6)	7751 (43.0)	10 278 (57.0)	5084 (28.2)	3313 (18.4)	1881 (10.4)	
60-69	10 296 (13.5)	4352 (42.3)	5944 (57.7)	2989 (29.0)	1901 (18.5)	1054 (10.2)	
≥70	6011 (7.9)	2719 (45.2)	3292 (54.8)	1645 (27.3)	1082 (18.0)	565 (9.4)	
Sex							
Male	40 225 (52.6)	17 255 (42.9)	22 970 (57.1)	11 480 (28.6)	7327 (18.2)	4163 (10.4)	<.001
Female	36 237 (47.4)	9888 (27.3)	26 349 (72.7)	15 009 (41.4)	7937 (21.9)	3403 (9.4)	
Psychiatric illness							
Substance use disorder	21 313 (27.9)	12 494 (58.6)	8819 (41.4)	4226 (19.8)	2763 (13.0)	1830 (8.6)	<.001
Schizophrenia	17 608 (23.0)	4330 (24.6)	13 278 (75.4)	6568 (37.3)	4384 (24.9)	2326 (13.2)	
Bipolar disorder	6871 (9.0)	1053 (15.3)	5818 (84.7)	3400 (49.5)	1797 (26.2)	621 (9.0)	
Depression	15 018 (19.6)	3323 (22.1)	11 695 (77.9)	6886 (45.8)	3493 (23.3)	1316 (8.8)	
Anxiety disorder	10 964 (14.3)	3987 (36.4)	6977 (63.6)	4101 (37.4)	1970 (18.0)	906 (8.3)	
Other mental disorders	4688 (6.1)	1956 (41.7)	2732 (58.3)	1308 (28.0)	857 (18.3)	567 (12.1)	
Health insurance type							
Health insurance	63 437 (83.0)	21 707 (34.2)	41 730 (65.8)	23 187 (36.6)	12 839 (20.2)	5704 (9.0)	<.001
Medicaid	13 025 (17.0)	5436 (41.7)	7589 (58.3)	3302 (25.5)	2425 (18.6)	1862 (14.3)	
Charlson comorbidity index							
0	28 244 (36.9)	9502 (33.6)	18 742 (66.4)	10 010 (35.5)	5907 (20.9)	2825 (10.0)	<.001
1	21 251 (27.8)	7147 (33.6)	14 104 (66.4)	7821 (36.8)	4239 (20.0)	2044 (9.6)	
2	12 140 (15.9)	4438 (37.0)	7702 (63.4)	4103 (33.8)	2424 (20.0)	1175 (9.7)	
≥3	14 827 (19.4)	6056 (40.8)	8771 (59.2)	4555 (30.7)	2694 (18.2)	1522 (10.3)	
Previous outpatient care							
Yes	55 989 (73.2)	14 824 (26.5)	41 165 (73.5)	22 196 (39.6)	12 694 (22.7)	6275 (11.2)	<.001
No	20 473 (26.8)	12 319 (60.2)	8154 (39.8)	4293 (21.0)	2570 (12.6)	1291 (6.3)	

Abbreviation: NA, not applicable.

### Timing of First Outpatient Follow-Up and Risk of Suicide

Table 2 shows the suicide rate per 100 000 person-years based on the timing of outpatient follow-up care after psychiatric inpatient discharge. The mean (SD) follow-up period was 30.8 (20.2) months. The suicide rate was 783.1 (95% CI, 744.1-822.1) per 100 000 person-years (1536 patients died of suicide during the study period); and among those with psychiatric illness, the suicide rate of patients with depression was reported as 1139.2 (95% CI, 1037.8-1240.7) per 100 000 person-years, which was higher than for other diagnosis groups.

**Table 2.** Suicide Rate Per 100 000 Person-Years After Psychiatric Discharge

Variables	At risk for suicide, person-years	Suicides during follow-up period, No.	Suicide rate per 100 000 person-years (95% CI)
Any psychiatric disorder	196 145.3	1536	783.1 (744.1-822.1)
Substance use disorder	50 404.8	411	815.4 (736.9-893.9)
Schizophrenia	38 503.3	296	768.8 (681.5-856.0)
Bipolar disorder	17 686.9	136	768.9 (640.2-897.7)
Depression	42 046.3	479	1139.2 (1037.8-1240.7)
Anxiety disorder	35 754.1	159	444.7 (375.7-513.7)
Other mental disorders	11 749.9	55	468.1 (344.7-591.5)

Table 3 depicts the association between the timing of the first outpatient follow-up and the risk of suicide. The HR for suicide risk in patients who had outpatient follow-up visits within 7 days compared with patients who did not have follow-up visits after discharge was 0.82 (95% CI, 0.80–0.83). The HR for suicide risk in patients who had follow-up visits within 8 to 14 days was 0.83 (0.95% CI, 0.82-0.85), and for 15 to 30 days it was 0.86 (95% CI, 0.84-0.89).

**Table 3.** Timing of First Outpatient Follow-Up After Psychiatric Discharge and Suicide Risk

Variables	HR (95% CI)						
	Any psychiatric disorder (N = 76 462)	Substance use disorder (n = 21 313)	Schizophrenia (n = 17 608)	Bipolar disorder (n = 6871)	Depression (n = 15 018)	Anxiety disorder (n = 10 964)	Other mental disorders (n = 4688)
Timing of first outpatient care after discharge							
No visit	1 [Reference]	1 [Reference]	1 [Reference]	1 [Reference]	1 [Reference]	1 [Reference]	1 [Reference]
≤7 d	0.83 (0.80-0.83)	0.92 (0.87-0.95)	0.55 (0.52-0.57)	0.67 (0.63-0.72)	0.87 (0.83-0.91)	1.04 (0.99-1.09)	0.80 (0.74-0.86)
8-14 d	0.83 (0.82-0.85)	1.00 (0.96-1.05)	0.56 (0.53-0.58)	0.68 (0.63-0.74)	0.87 (0.83-0.91)	1.04 (0.99-1.11)	0.77 (0.71-0.84)
15-30 d	0.86 (0.84-0.89)	1.00 (0.95-1.05)	0.57 (0.54-0.60)	0.73 (0.65-0.80)	0.97 (0.91-1.03)	1.00 (0.93-1.07)	0.84 (0.76-0.92)
Psychiatric illness							
Substance use disorder	1 [Reference]	NA	NA	NA	NA	NA	NA
Schizophrenia	1.21 (1.18-1.23)	NA	NA	NA	NA	NA	NA
Bipolar disorder	1.05 (1.03-1.09)	NA	NA	NA	NA	NA	NA
Depression	0.90 (0.88-0.92)	NA	NA	NA	NA	NA	NA
Anxiety disorder	0.74 (0.72-0.75)	NA	NA	NA	NA	NA	NA
Other mental disorders	0.94 (0.91-0.98)	NA	NA	NA	NA	NA	NA
Age at discharge	1.00 (1.00-1.00)	1.00 (1.00-1.00)	1.00 (1.00-1.00)	1.00 (1.00-1.00)	1.00 (1.00-1.00)	1.00 (1.00-1.00)	1.00 (1.00-1.00)
Sex							
Male	1 [Reference]	1 [Reference]	1 [Reference]	1 [Reference]	1 [Reference]	1 [Reference]	1 [Reference]
Female	0.89 (0.88-0.91)	0.74 (0.72-0.77)	0.96 (0.94-0.99)	1.02 (0.97-1.07)	0.93 (0.90-0.96)	0.90 (0.86-0.94)	0.98 (0.93-1.04)
Type of health insurance							
Health insurance	1 [Reference]	1 [Reference]	1 [Reference]	1 [Reference]	1 [Reference]	1 [Reference]	1 [Reference]
Medicaid	1.33 (1.30-1.35)	1.28 (1.23-1.33)	1.33 (1.28-1.38)	1.35 (1.25-1.46)	1.23 (1.17-1.30)	1.17 (1.10-1.26)	1.47 (1.38-1.57)
Charlson comorbidity index							
0	1 [Reference]	1 [Reference]	1 [Reference]	1 [Reference]	1 [Reference]	1 [Reference]	1 [Reference]
1	0.99 (0.97-1.01)	1.04 (1.00-1.08)	0.96 (0.93-1.00)	1.00 (0.95-1.06)	0.99 (0.95-1.03)	0.99 (0.94-1.04)	0.95 (0.89-1.02)
2	1.01 (0.99-1.04)	1.06 (1.02-1.11)	0.97 (0.92-1.02)	1.02 (0.95-1.10)	1.02 (0.97-1.08)	0.99 (0.94-1.05)	1.00 (0.91-1.09)
≥3	1.01 (0.99-1.03)	1.06 (1.02-1.10)	0.96 (0.91-1.02)	1.04 (0.95-1.13)	1.03 (0.98-1.08)	0.99 (0.93-1.05)	1.03 (0.93-1.13)
Previous outpatient care							
No	1 [Reference]	1 [Reference]	1 [Reference]	1 [Reference]	1 [Reference]	1 [Reference]	1 [Reference]
Yes	1.14 (1.12-1.16)	1.15 (1.12-1.18)	1.14 (1.10-1.19)	1.08 (1.01-1.15)	1.11 (1.06-1.17)	1.13 (1.08-1.18)	1.20 (1.11-1.29)

Abbreviations: HR, hazard ratio; NA, not applicable.

Similar results were found for those with schizophrenia and bipolar disorder. Compared with patients who did not have follow-up visits within 30 days after discharge, those who did, especially those who had early follow-up visits, had a lower risk of suicide. However, for those with substance use disorder, only the suicide risk of patients who visited within 7 days after discharge was significantly lower compared with patients who did not have any follow-up visits (HR, 0.92 [95% CI, 0.89-0.95]). Moreover, patients with depression whose first follow-up visit occurred within 7 days or 8 to 14 days had a lower risk of suicide compared with those who did not have any follow-up visits, but patients who visited within 15 to 30 days had no significantly reduced risk of suicide. There was no significant association between outpatient follow-up visits after discharge and suicide in anxiety disorder.

## Discussion

Early follow-up care after discharge from a psychiatric hospital is strongly recommended to prevent suicide. This population-based study examined the association between the timing of outpatient follow-up after discharge and risk of suicide, finding that earlier outpatient care was significantly associated with lower risk of suicide for patients with psychiatric illness except for anxiety disorder.

In this study, the rate of receiving outpatient care within 7 days and 30 days were 34.7% and 64.5%, respectively. These results were similar for each psychiatric illness; patients with bipolar disorder had the highest rate of receiving outpatient follow-up care among psychiatric illness. However, the rates in our study were low compared with other studies. In 2021, among patients aged 6 years and older under Commercial Health Maintenance Organization in the US, the rate of follow-up within 7 days of discharge was 48.0% and within 30 days was 70.6%.^15^ In a Japanese cohort study of Medicaid patients with schizophrenia and bipolar disorder, 85.1% of patients had follow-up visits within 30 days after discharge.^10^ The low rate in our study may be attributed to the mental health delivery system of Korea, which has focused on hospital care rather than outpatient care. This is also supported by the fact that the rate of readmission within 30 days after discharge was 6.1% in our study. In addition, patients not only experiencing adverse effects and poor therapeutic alliance but also facing stigma may have contributed to no follow-up visits after discharge.^22^

There was a significant association between early outpatient care after discharge and risk of suicide. To the best of our knowledge, no previous study has examined the association between outpatient follow-up with short timelines and long-term suicide risk for adult patients. Nevertheless, these findings were consistent with those of previous studies among other study populations that used different follow-up timelines to examine their association with suicide, indicating that early outpatient follow-up after discharge reduces the risk of suicide. A previous Korean study, in which outpatient follow-up visits occurred within 1 year after discharge from hospitals for psychiatric illness, found that patients who received 4 or more outpatient visits after discharge had a lower suicide risk than those who did not receive any (adjusted HR, 0.53 [95% CI, 0.30-0.93]).^23^ A case-control study of patients aged 18 to 65 years in England found that 120 patients died by suicide within 2 weeks after discharge from psychiatric hospitals, with nearly half of the suicides occurring before the first outpatient appointment.^24^ In a US study, 56.5% of youths aged 10 to 18 years received mental health follow-up care within 7 days after discharge from psychiatric hospitals, which was significantly associated with a lower risk of suicide after discharge (relative risk, 0.44, [95% CI, 0.23-0.83]).^12^ These findings support the existing quality indicators^14^ and the need for transitions from discharge to outpatient follow-up care. In this study, patients with substance use disorder, schizophrenia, bipolar disorder, and depression had a lower risk of suicide when they received outpatient follow-up care within 7 days. These results also support the NICE guidelines, which recommend a follow-up period of within 7 days after discharge.^18^ In Korea, the clinical practice guideline for depression recommends follow-up care at 1 month intervals, as the average improvement period after taking antidepressants is 13 days, a period of at least 2 weeks is required to evaluate the treatment effect.^25^

The rates of receiving outpatient follow-up care within 7 days of discharge for substance use disorder and schizophrenia were 19.8% and 37.3%, respectively, which were the lowest rates among psychiatric illness, and early follow-up was found to be significantly associated with lower risk of suicide. For anxiety disorder, however, there was no significant association between outpatient follow-up and the risk of suicide, while the rate of receiving outpatient follow-up care within 7 days was similar to that of schizophrenia. There is controversy about whether anxiety disorders are associated with suicide and suicide attempt.^26,27^ Moreover, general anxiety disorder often coexists with other psychiatric illness that may affect a patient’s health outcome.^28^ The results suggest that other factors apart from postdischarge outpatient visits may have a role to play in suicide risk. Further studies are needed to consider potential factors that may be associated with long-term suicide risk.

### Limitations

This study has limitations. First, psychiatric illnesses were extracted only through *ICD-10* codes because no information regarding functional status was available in the NHICD. The diagnostic accuracy relies on the clinical judgement of clinicians or coding practice. This study could not confirm the diagnostic accuracy through retrospective medical record reviews that are used in studies using claims data because patient consent is required for medical record review. However, in previous studies of Korea that investigated the diagnostic consistency between the NHICD and electronic medical record, psychiatric illness was higher than for other diseases^29^ and highly sensitive of disease because of stigma against psychiatric illness in Korea.^30^ Second, important factors that may affect suicide risk, such as social support, history of suicide attempts, and socioeconomic status, were not considered as potential covariates. However, this information is not available because the NHICD is collected for reimbursement. Further research is required considering the factors affecting suicide. Third, the results of this study suggest that early follow-up outpatient care is associated with reduced risk of suicide after discharge; however, the timing of outpatient care is inevitably different depending on cost efficiency, institutional priorities, and patient-centeredness.^31^ Therefore, the results should be interpreted in consideration of these points. Additionally, Korea’s mental health system is admission-based care with a long length of hospital stay.^32^ As a result, there is a possibility that the risk of suicide may be underestimated, so it is necessary to consider the health system when interpreting the study results. Nevertheless, to our knowledge, this study was the first to investigate the association between the timing of the first outpatient follow-up and the risk of suicide after discharge using a nationwide population-based database.

## Conclusions

In this population-based retrospective cohort study including all patients with psychiatric illness, early outpatient follow-up visits after discharge were found to be associated with lower risk of suicide. In particular, a significant association between outpatient treatment and suicide risk within 7 days after discharge was found in patients with substance use disorder, schizophrenia, bipolar disorder, and depression. These findings suggest that more intensive follow-up during the period immediately after discharge is needed to prevent suicide among patients with psychiatric illness, especially those who are deemed to be at high risk for suicide during admission.

## Notes

Supplement.
Data Sharing Statement
