# Supplementary material for: Follow-Up Timing After Discharge and Suicide Risk Among Patients Hospitalized With Psychiatric Illness
Source: JAMA Netw Open. 2023 Oct 9;6(10):e2336767. doi: 10.1001/jamanetworkopen.2023.36767 (PMC10562943; doi:10.1001/jamanetworkopen.2023.36767)
Supplement: Supplement. — Data Sharing Statement [file jamanetwopen-e2336767-s001.pdf]

Data Sharing Statement

Che. Follow-Up Timing After Discharge and Suicide Risk Among Patients Hospitalized With Psychiatric Illness. *JAMA Netw Open*. Published October 09, 2023.
doi:10.1001/jamanetworkopen.2023.36767

Data
Data available: No

Additional Information
Explanation for why data not available: The datasets generated and/or analyzed during the current study are not publicly available due to HIRA's policy.
